# Supplementary material for: Efficient artificial mineralization route to decontaminate Arsenic(III) polluted water - the Tooeleite Way
Source: Sci Rep. 2016 May 18;6:26031. doi: 10.1038/srep26031 (PMC4870689; doi:10.1038/srep26031)

Supplementary Information

Efficient artificial mineralization route to decontaminate Arsenic(III) polluted water - the Tooeleite Way

Authors: Arindam Malakar[1], Bidisa Das[2], Samirul Islam[1], Carlo Meneghini[3], Giovanni De Giudici[4], Marco Merlini[5], Yury V. Kolen'ko[6], Antonella Iadecola[7], Giuliana Aquilanti[7], Somobrata Acharya[2], and Sugata Ray[1,2,*]

Affiliations:

[1]Department of Materials Science, Indian Association for the Cultivation of Science, Jadavpur, Kolkata 700032, India

[2]Centre for Advanced Materials, Indian Association for the Cultivation of Science, Jadavpur, Kolkata 700032, India

[3]Dipartimento di Scienze, Universitá Roma Tre, Via della Vasca Navale, 84 I-00146 Roma, Italy

[4]Department of Chemical and Geological Sciences, University of Cagliari, 09127 Cagliari, Italy

[5]Universita di Milano–Dip. di Scienze della Terra Ardito Desio, Milano, Italy

[6]International Iberian Nanotechnology Laboratory, Av. Mestre José Veiga s/n, 4715-330 Braga, Portugal

[7]Elettra-Sincrotrone Trieste S.C.p.A., Strada Statale 14,km 163.5, 34149 Basovizza, Trieste, Italy

**Correspondence to: mssr@iacs.res.in*

Pages: 1-6

Tables: S1-S3

Figures: S1-S3

Table S1 Shows arsenic and iron content of different samples and their genesis.

Sample Name	Genesis	Fe (mg/L)	As (mg/L)
NW-Kolkata	Water collected from the bore well of Dhapdhapi, Baruipur, West Bengal in summer	0.663	0.392
NW-Kolkata_Blank	Filtrate after treating NW-Kolkata without ZnS	0.045	0.252
NW-Kolkata_ZnS-NR	Filtrate after treating NW-Kolkata with ZnS	0.067	0.027
AF-Lab	Laboratory make solution of Fe^{3+} and As^{3+} having same concentration as NW-Kolkata	0.667	0.392
AF-Lab_Blank	Filtrate after treating AF-Lab without ZnS	0.101	0.350
AF-Lab_ZnS-NR	Filtrate after treating AF-Lab with ZnS	0.193	0.018
NW-Sardinia	Natural water collected from Iglesius, having identical aqueous chemistry as BaccuLocci mine area sans As, which has then been spiked externally with As equivalent of Baccu Locci	0.238	0.198
NW-Sardinia_Blank	Filtrate after treating NW-Sardinia without ZnS	0.055	0.108
NW-Sardinia_ZnS-NR	Filtrate after treating NW-Sardinia with ZnS	0.001	0.019
NW-Kolkata (July)	Water collected from the bore well of Dhapdhapi, Baruipur, West Bengal in rainy season	0.457	0.148

Table S2 Structural parameters obtained after Rietveld refinement of samples compared with Tooeleite data from the literature[18].

Sample	a (Å)	b (Å)	c (Å)	beta (deg)	Vol (Å^3)
**NW-Kolkata_ZnS-NR**	8.9238(7)	6.4102(9)	9.5159(4)	97.681(8)°	539.47(1)
**AF-Lab_ZnS-NR**	8.9378(5)	6.4330(1)	9.5760(4)	97.737(9)°	545.58(4)
**Tooeleite**	8.9575(1)	6.4238(1)	9.7912(1)	96.032(1)°	560.27(3)

Table S3 Summary of structural parameters and agreement factors after Rietveld refinement

Crystal data

Space group *C* 1 2/m 1 (no. 12)

Unit cell dimensions

a = 8.9348 Å

b = 6.4291 Å

c = 9.5827 Å

β = 97.74 °

Cell volume 545.44 Å^3

Atomic coordinates and isotropic displacement parameters (in Å ^{2})

Atom	Wyck.	x	y	z
As1	4*i*	0.7768(9)	0	0.2040(7)
Fe1	4*i*	0.1378(12)	0	0.1232(8)
Fe2	2*b*	1/2	0	0
O1	4*i*	0.331(3)	0	0.102(3)
O2	4*i*	0.187(3)	0	0.346(3)
O3	4*i*	0.890(3)	0	0.051(3)
O4	8*j*	0.624(2)	0.186(2)	0.161(2)
O5	2*d*	1/2	0	1/2

R factors : [668=668+0/25]

R(obs)= 2.28 wR(obs)= 2.60 R(all)= 2.28 wR(all)= 2.60

Profile R factors :[1900/25+25], Damping factor: 0.5000

GOF = 0.60 Rp = 2.14 wRp = 3.25

Fig. S1 Observed (blue), calculated (red) and difference (black) XRPD curve, with marks for Bragg peaks.

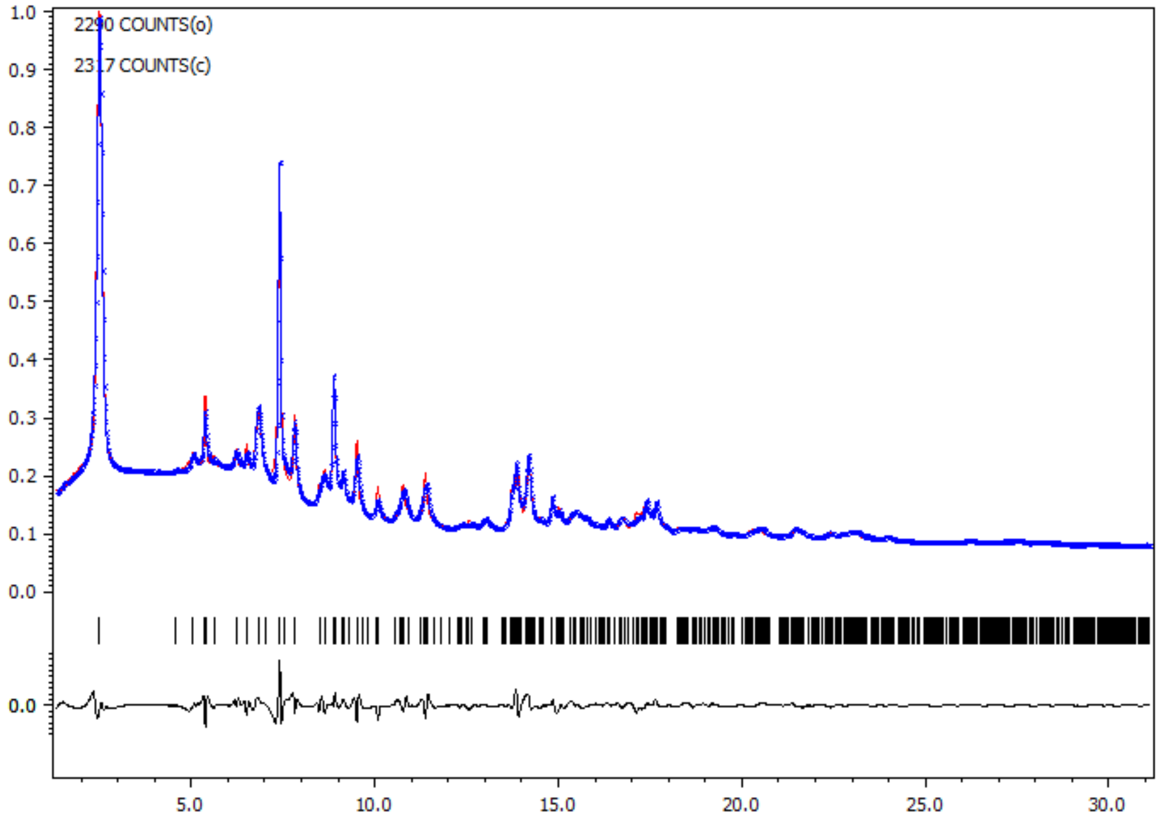

Fig. S2 (a) Photoemission spectra of Fe 2*p*, (b) Fe *K*-edge XANES obtained from AF-Lab_ZnS-NR and NW-Kolkata_ZnS-NR (c) Photoemission spectra of As 3*d* from samples AF-Lab_ZnS-NR, NW-Kolkata_ZnS-NR and NW-Sardinia_ZnS-NR. EXAFS fitting of (d) Fe *K*-edges with different components obtained from sample AF-Lab_ZnS-NR only.

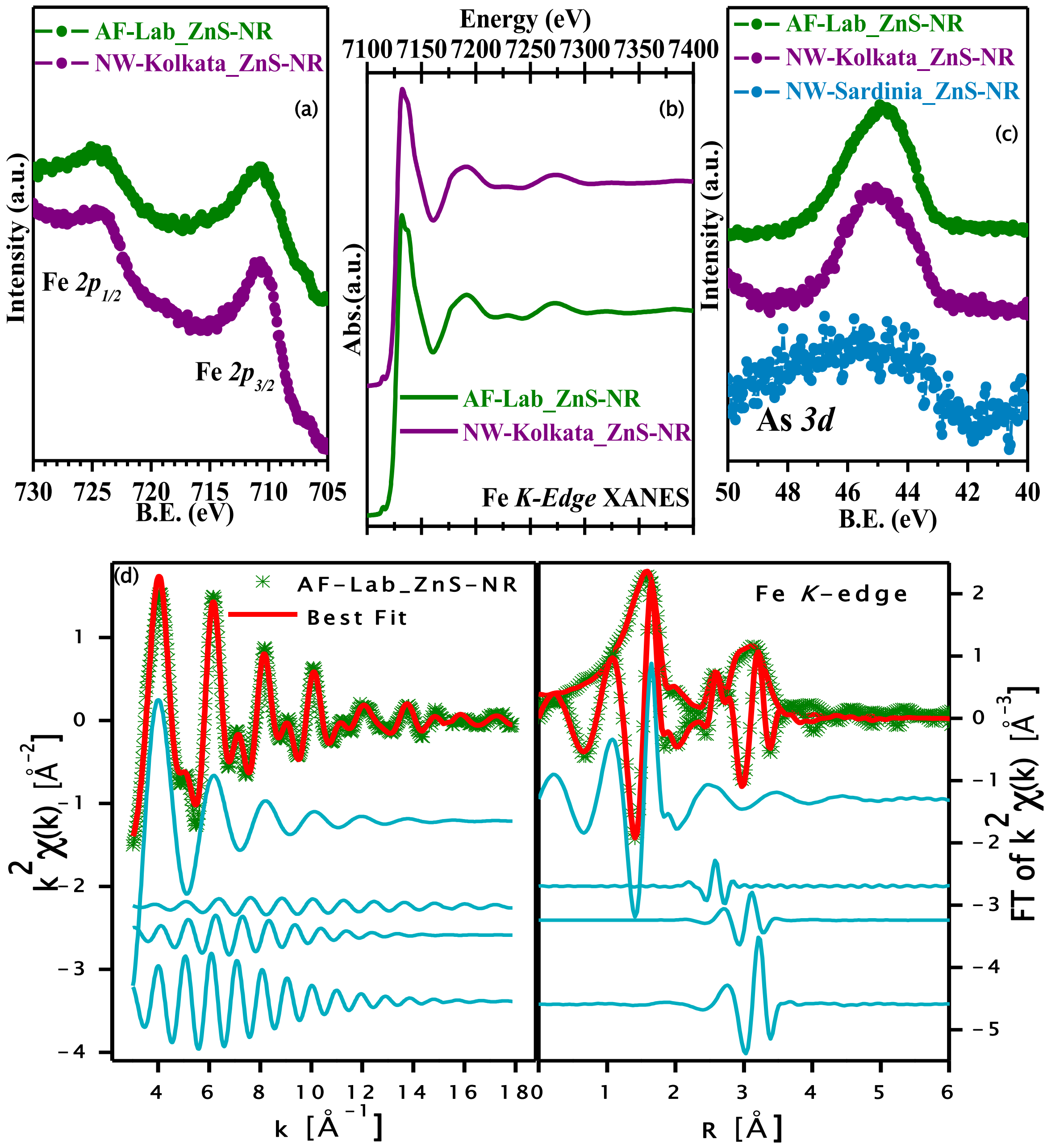

Fig. S3 (a) Extended crystal structure of Tooeleite. Consider the portion marked inside the black box. The upper two and lower two As (green) atoms are bonded to the chain, the middle two As atoms are in different plane (b) The marked portion, BFe8As4, is longitudinally extended structure of BFe4As4 when two B molecules interact with it as shown in (c). In this way infinitely long chains can form. These chains then interact laterally to give the crystalline structure shown in (a).

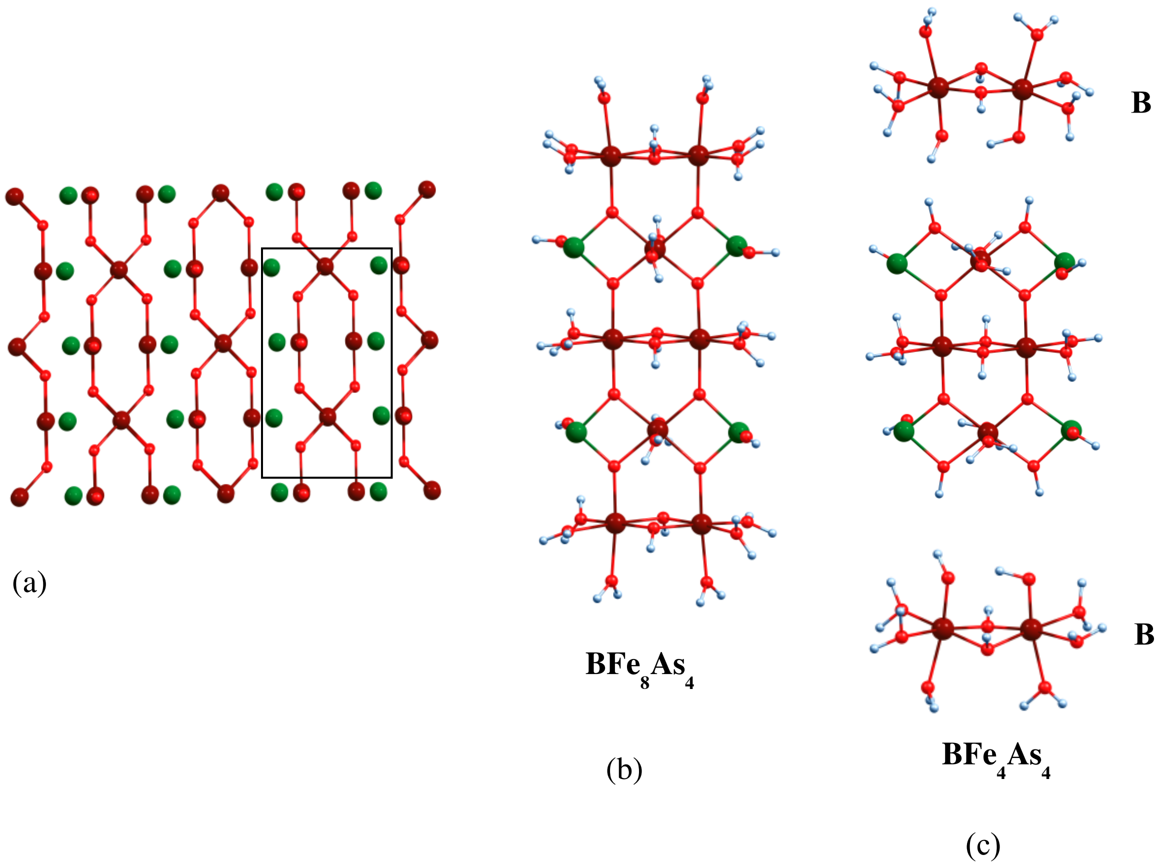

Supplement: Supplementary Information [file srep26031-s1.pdf]
